# Animal Models of Neurological Disorders: Where Are We Now?

**DOI:** 10.3390/biomedicines11051253

**Published:** 2023-04-23

**Authors:** Sandesh Panthi, Marc Ekker

**Affiliations:** 1 Department of Psychiatry, School of Clinical Sciences, Monash University, Clayton, VIC 3168, Australia; 2 Department of Biology, Faculty of Science, University of Ottawa, Ottawa, ON K1N 6N5, Canada

The Special Issue “Animal Models of Neurological Disorders: Where Are We Now?”, published in *Biomedicines*, was intended to cover original articles and reviews on every aspect of mammalian and non-mammalian animal models of various neurological disorders. We received high-quality research articles; among them, four original research articles, three review articles and one systematic review were published. Canta et al. compared the long-term course of diabetic polyneuropathy in streptozotocin-treated Sprague Dawley and Zucker diabetic fatty rats using various behavioural, immunological and biochemical tests [1]. Vaz et al. established a zebrafish model of de novo missense variant in pyruvate dehydrogenase kinase 1(PDK1), which was found in a human patient with dysmorphic facial features, severe developmental delay and seizures. They were able to characterize phenotypes resulting from the expression of mutant PDK1 in vivo, which is not possible when using patient fibroblasts [2]. In the third research article published in this Special Issue, Hatat et al. examined the effect of two compounds (Fluoxetine and Acacetin) in a rat model of unilateral peripheral vestibulopathy [3]. In another study, Quelle-Regaldie et al. characterized a zebrafish model of NOP56 loss of function. NOP56 is part of a C/D box small nucleolar ribonucleoprotein complex responsible for the cleavage and modification of precursor ribosomal RNAs and the assembly of the 60S ribosomal subunit. The authors aimed at establishing a better understanding of NOP56’s role in the normal functioning and development of the central nervous system. They used various innovative methods and found that NOP56 loss of function causes a severe neurodegenerative phenotype characterized by early death, increased apoptosis, absence of cerebellum, a reduced numbers of spinal cord neurons and impaired movement [4]. Tighilet et al. reviewed animal models of peripheral vestibulopathies of different types and stages of vestibular pathologies [5]. Garbuz et al. reviewed rodent models of audiogenic epilepsy, where they highlighted in depth the genetic aspects, current problems and advantages of using of rodent strains predisposed to audiogenic epilepsy in current epileptology [6]. Guidetti et al. reviewed the current literature on intracerebral recordings of direct current stimulations and the electric fields induced by transcranial alternating current stimulations in humans and non-human primates [7]. Zhang et al. systemically reviewed 51 published studies of genetic rodent models of Parkinson’s Disease. This review aimed at answering if there are reproducible non-motor phenotypes among genetic Parkinson’s Disease rodent models, as well as whether the phenotypes are age-dependent and if they are translatable. This review highlighted the importance of phenotypic assay choice and robust experimental design in improving phenotype reproducibility [8].

The use of animal models in the investigation of neurological diseases and disorders is a powerful method to uncover key principles and mechanisms. We have benefited from animal models by conducting various kinds of experiments and studying mechanisms underlying diseases and disorders in ways that would be impossible and unthinkable to do with human subjects. Predictive validity, symptoms, similarity to human conditions and tractability are all factors that influence the usefulness of an animal model. This first edition of this Special Issue in Biomedicines has covered various aspect of different types of neurological disorders, and we are happy to share that a second issue will be launched soon.
